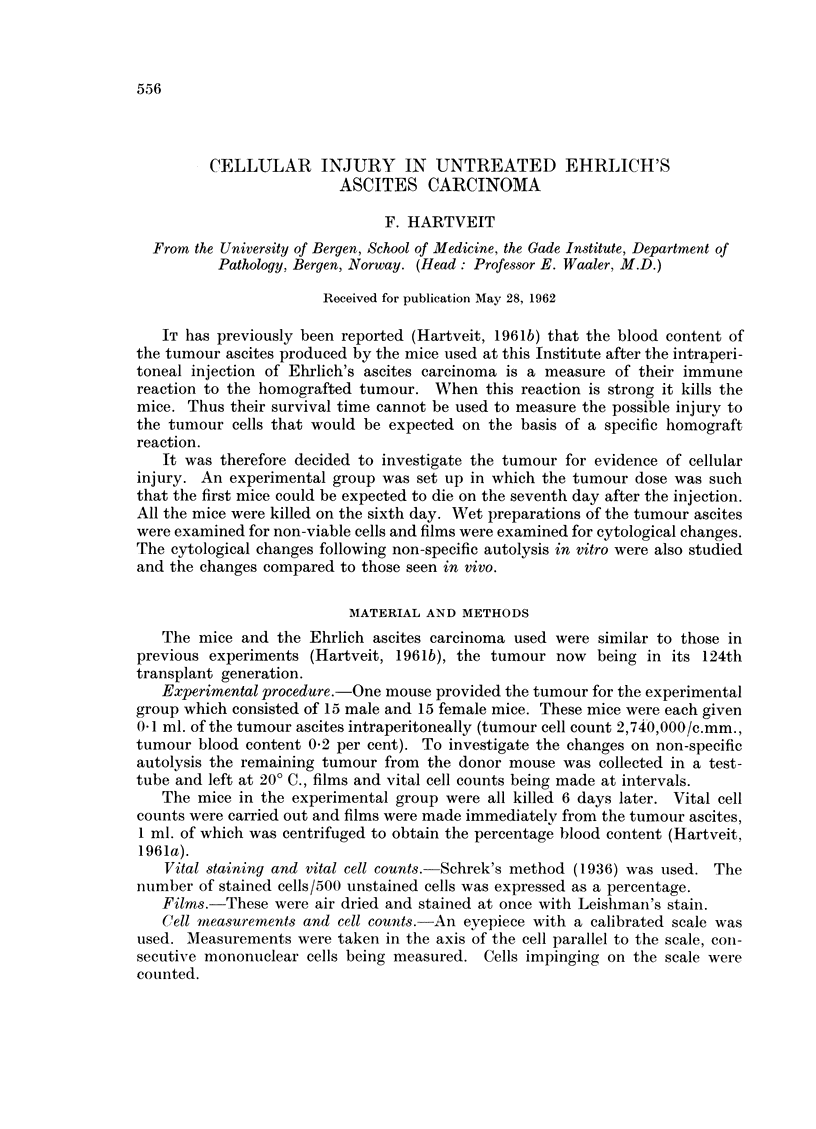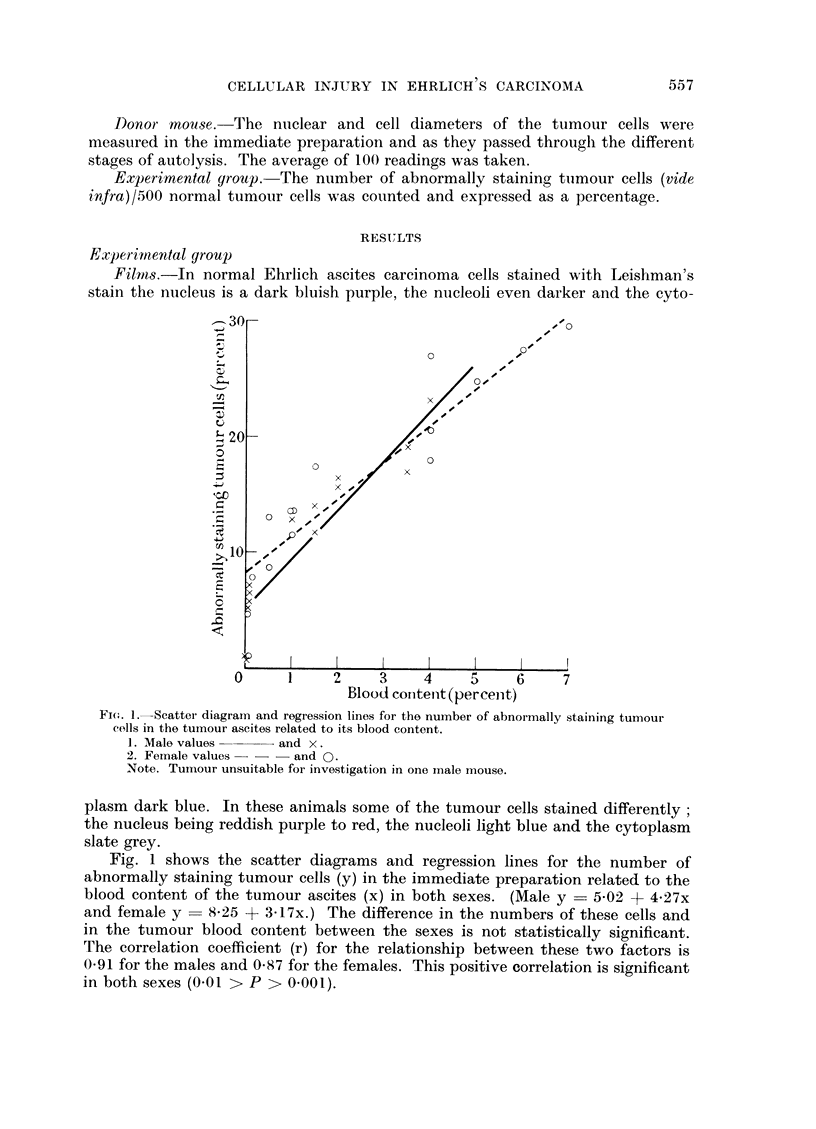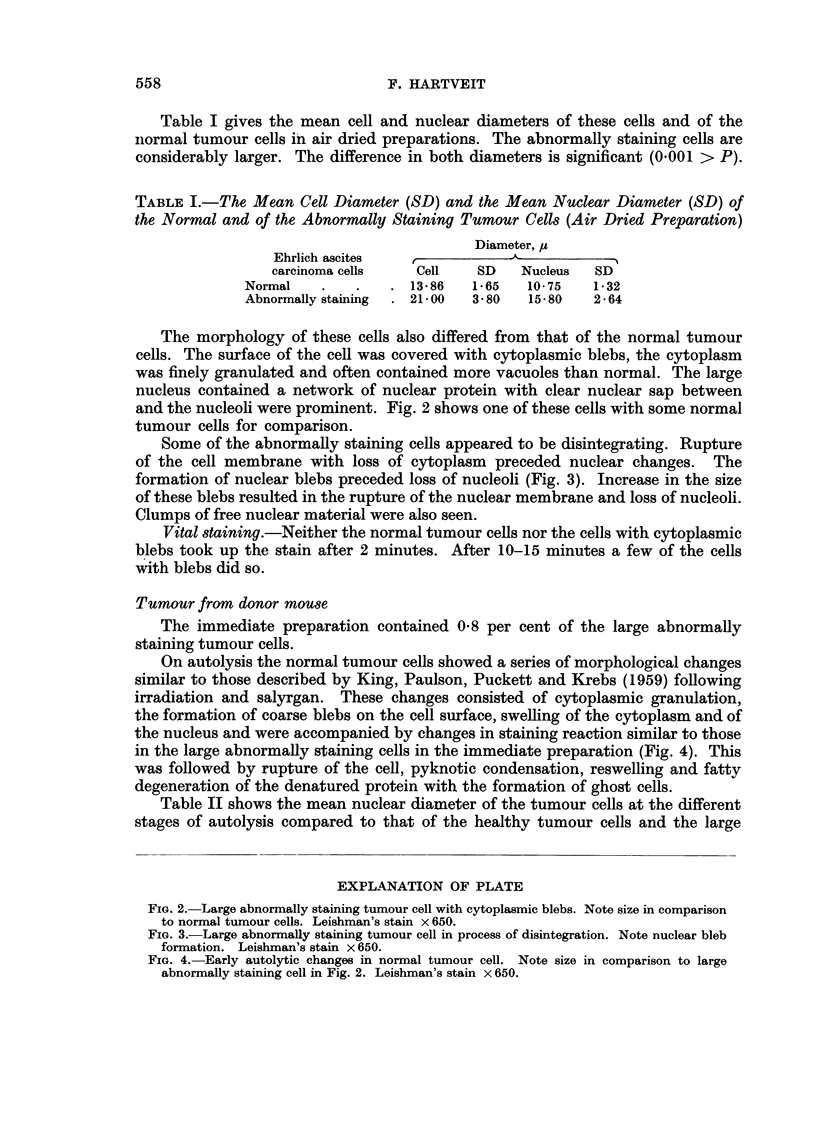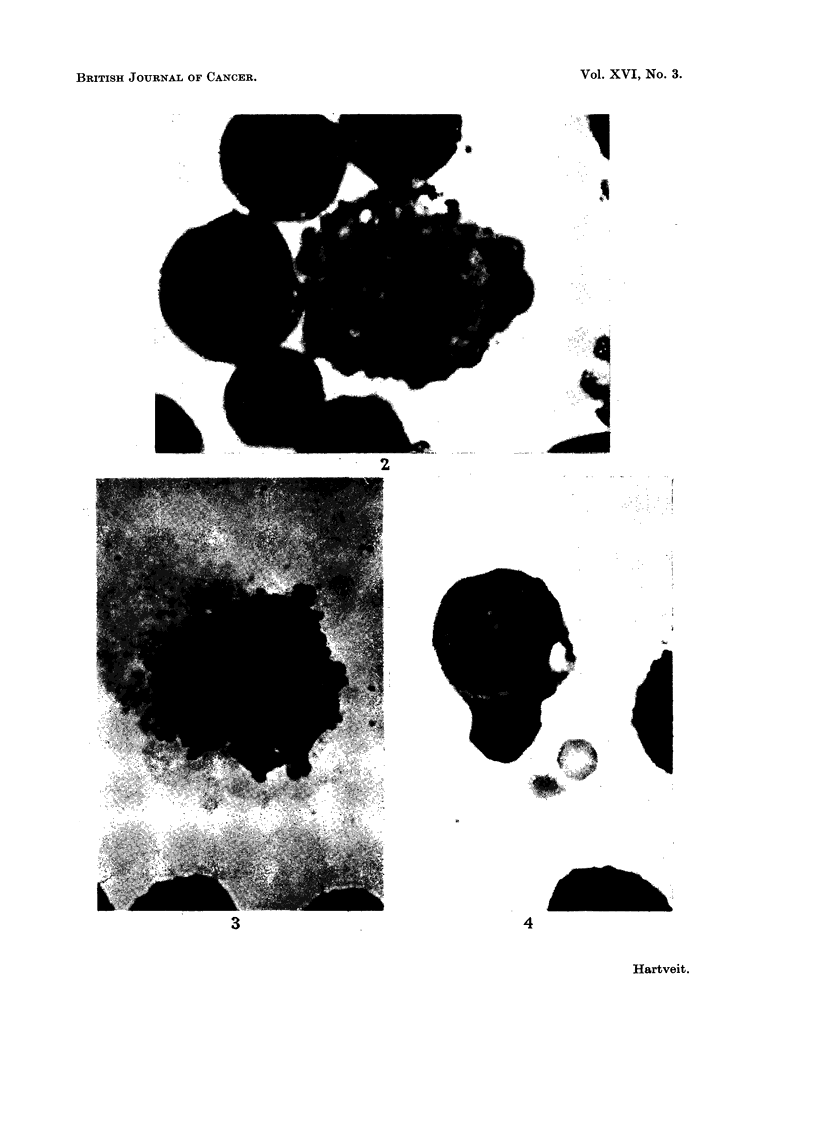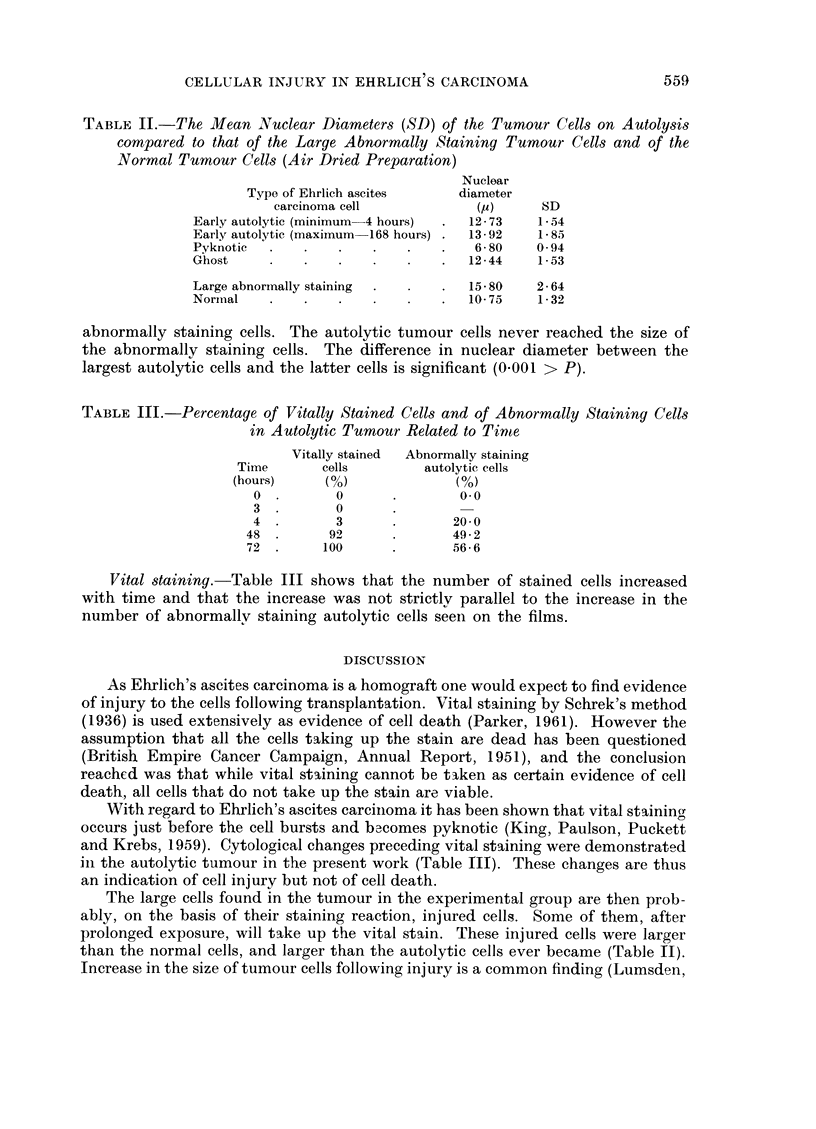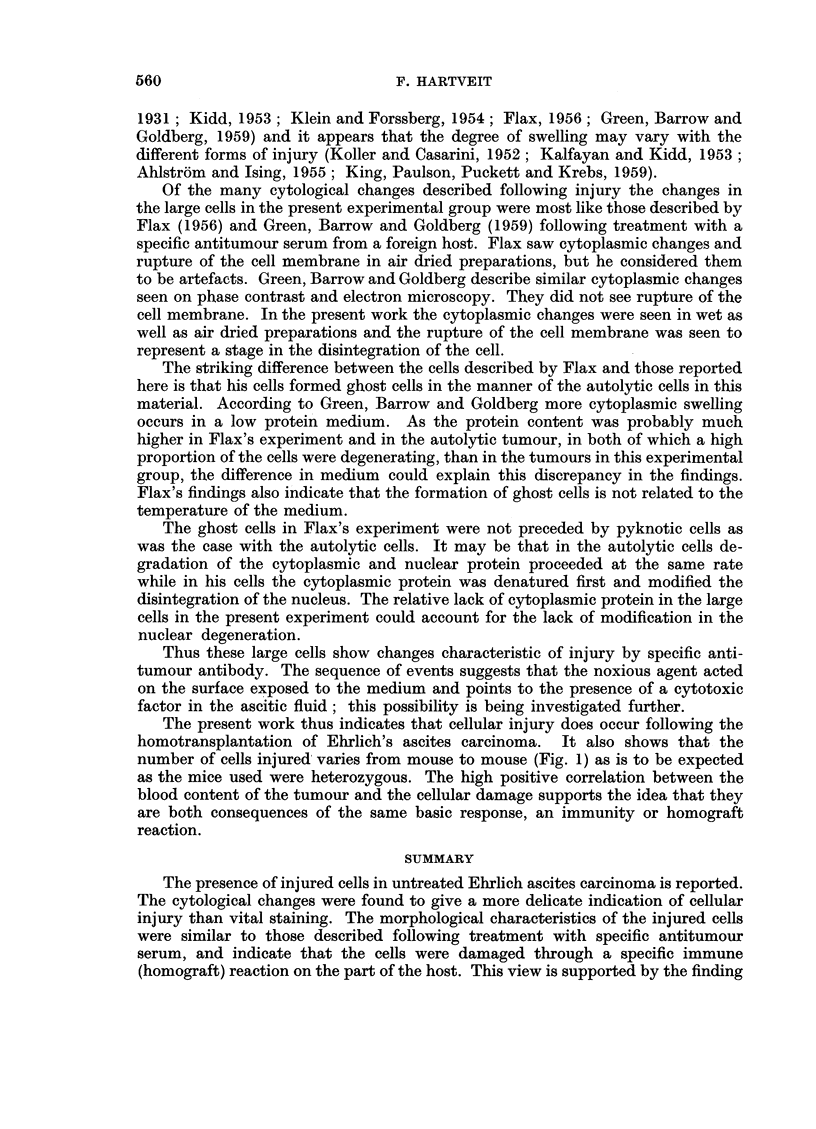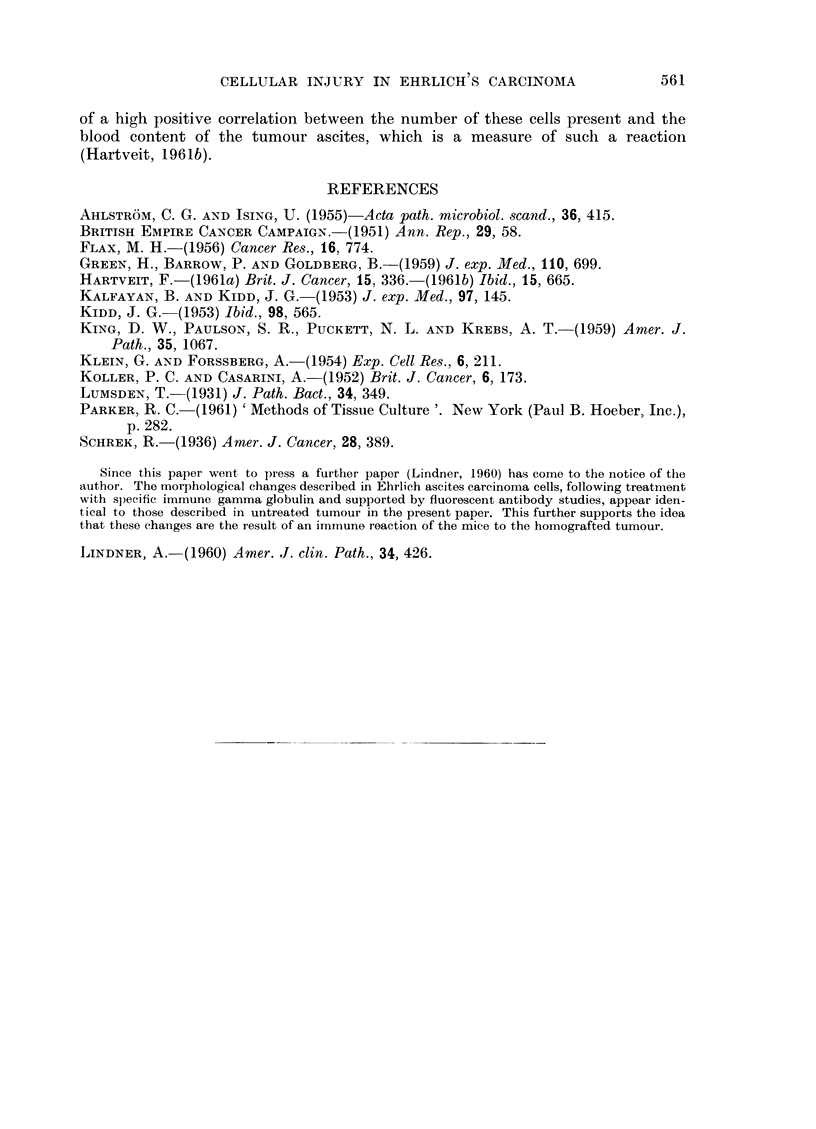# Cellular Injury in Untreated Ehrlich's Ascites Carcinoma

**DOI:** 10.1038/bjc.1962.64

**Published:** 1962-09

**Authors:** F. Hartveit

## Abstract

**Images:**


### CELLULAR INJURY IN UNTREATED EHRLICH'S ASCITES CARCINOMA

F. HARTVEIT

*From the University of Bergen, School of Medicine, the Gade Institute, Department of Pathology, Bergen, Norway. (Head : Professor E. Waaler, M.D.)*

Received for publication May 28, 1962

It has previously been reported (Hartveit, 1961*b*) that the blood content of the tumour ascites produced by the mice used at this Institute after the intraperitoneal injection of Ehrlich's ascites carcinoma is a measure of their immune reaction to the homografted tumour. When this reaction is strong it kills the mice. Thus their survival time cannot be used to measure the possible injury to the tumour cells that would be expected on the basis of a specific homograft reaction.

It was therefore decided to investigate the tumour for evidence of cellular injury. An experimental group was set up in which the tumour dose was such that the first mice could be expected to die on the seventh day after the injection. All the mice were killed on the sixth day. Wet preparations of the tumour ascites were examined for non-viable cells and films were examined for cytological changes. The cytological changes following non-specific autolysis *in vitro* were also studied and the changes compared to those seen *in vivo.*

### MATERIAL AND METHODS

The mice and the Ehrlich ascites carcinoma used were similar to those in previous experiments (Hartveit, 1961*b*), the tumour now being in its 124th transplant generation.

*Experimental procedure.*—One mouse provided the tumour for the experimental group which consisted of 15 male and 15 female mice. These mice were each given 0·1 ml. of the tumour ascites intraperitoneally (tumour cell count 2,740,000/c.mm., tumour blood content 0·2 per cent). To investigate the changes on non-specific autolysis the remaining tumour from the donor mouse was collected in a test-tube and left at 20° C., films and vital cell counts being made at intervals.

The mice in the experimental group were all killed 6 days later. Vital cell counts were carried out and films were made immediately from the tumour ascites, 1 ml. of which was centrifuged to obtain the percentage blood content (Hartveit, 1961*a*).

*Vital staining and vital cell counts.*—Schrek's method (1936) was used. The number of stained cells/500 unstained cells was expressed as a percentage.

*Films.*—These were air dried and stained at once with Leishman's stain.

*Cell measurements and cell counts.*—An eyepiece with a calibrated scale was used. Measurements were taken in the axis of the cell parallel to the scale, consecutive mononuclear cells being measured. Cells impinging on the scale were counted.

*Donor mouse.*—The nuclear and cell diameters of the tumour cells were measured in the immediate preparation and as they passed through the different stages of autolysis. The average of 100 readings was taken.

*Experimental group.*—The number of abnormally staining tumour cells (*vide infra*)/500 normal tumour cells was counted and expressed as a percentage.

RESULTS

*Experimental group*

*Films.*—In normal Ehrlich ascites carcinoma cells stained with Leishman's stain the nucleus is a dark bluish purple, the nucleoli even darker and the cyto-

FIG. 1.—Scatter diagram and regression lines for the number of abnormally staining tumour cells in the tumour ascites related to its blood content.
1. Male values ———— and ×.
2. Female values — — — and ○.
Note. Tumour unsuitable for investigation in one male mouse.

plasm dark blue. In these animals some of the tumour cells stained differently; the nucleus being reddish purple to red, the nucleoli light blue and the cytoplasm slate grey.

Fig. 1 shows the scatter diagrams and regression lines for the number of abnormally staining tumour cells (y) in the immediate preparation related to the blood content of the tumour ascites (x) in both sexes. (Male $y = 5{\cdot}02 + 4{\cdot}27x$ and female $y = 8{\cdot}25 + 3{\cdot}17x$.) The difference in the numbers of these cells and in the tumour blood content between the sexes is not statistically significant. The correlation coefficient (r) for the relationship between these two factors is 0·91 for the males and 0·87 for the females. This positive correlation is significant in both sexes ($0{\cdot}01 > P > 0{\cdot}001$).

Table I gives the mean cell and nuclear diameters of these cells and of the normal tumour cells in air dried preparations. The abnormally staining cells are considerably larger. The difference in both diameters is significant ($0 \cdot 001 > P$).

TABLE I.—*The Mean Cell Diameter (SD) and the Mean Nuclear Diameter (SD) of the Normal and of the Abnormally Staining Tumour Cells (Air Dried Preparation)*

| Ehrlich ascites carcinoma cells | Diameter, μ | | | |
|---|---|---|---|---|
| | Cell | SD | Nucleus | SD |
| Normal . . . | 13·86 | 1·65 | 10·75 | 1·32 |
| Abnormally staining . | 21·00 | 3·80 | 15·80 | 2·64 |

The morphology of these cells also differed from that of the normal tumour cells. The surface of the cell was covered with cytoplasmic blebs, the cytoplasm was finely granulated and often contained more vacuoles than normal. The large nucleus contained a network of nuclear protein with clear nuclear sap between and the nucleoli were prominent. Fig. 2 shows one of these cells with some normal tumour cells for comparison.

Some of the abnormally staining cells appeared to be disintegrating. Rupture of the cell membrane with loss of cytoplasm preceded nuclear changes. The formation of nuclear blebs preceded loss of nucleoli (Fig. 3). Increase in the size of these blebs resulted in the rupture of the nuclear membrane and loss of nucleoli. Clumps of free nuclear material were also seen.

*Vital staining.*—Neither the normal tumour cells nor the cells with cytoplasmic blebs took up the stain after 2 minutes. After 10–15 minutes a few of the cells with blebs did so.

*Tumour from donor mouse*

The immediate preparation contained 0·8 per cent of the large abnormally staining tumour cells.

On autolysis the normal tumour cells showed a series of morphological changes similar to those described by King, Paulson, Puckett and Krebs (1959) following irradiation and salyrgan. These changes consisted of cytoplasmic granulation, the formation of coarse blebs on the cell surface, swelling of the cytoplasm and of the nucleus and were accompanied by changes in staining reaction similar to those in the large abnormally staining cells in the immediate preparation (Fig. 4). This was followed by rupture of the cell, pyknotic condensation, reswelling and fatty degeneration of the denatured protein with the formation of ghost cells.

Table II shows the mean nuclear diameter of the tumour cells at the different stages of autolysis compared to that of the healthy tumour cells and the large

---

EXPLANATION OF PLATE

FIG. 2.—Large abnormally staining tumour cell with cytoplasmic blebs. Note size in comparison to normal tumour cells. Leishman's stain ×650.

FIG. 3.—Large abnormally staining tumour cell in process of disintegration. Note nuclear bleb formation. Leishman's stain ×650.

FIG. 4.—Early autolytic changes in normal tumour cell. Note size in comparison to large abnormally staining cell in Fig. 2. Leishman's stain ×650.

2

3

4

TABLE II.—*The Mean Nuclear Diameters (SD) of the Tumour Cells on Autolysis compared to that of the Large Abnormally Staining Tumour Cells and of the Normal Tumour Cells (Air Dried Preparation)*

| Type of Ehrlich ascites carcinoma cell | Nuclear diameter ($\mu$) | SD |
|---|---|---|
| Early autolytic (minimum—4 hours) | 12·73 | 1·54 |
| Early autolytic (maximum—168 hours) | 13·92 | 1·85 |
| Pyknotic | 6·80 | 0·94 |
| Ghost | 12·44 | 1·53 |
| Large abnormally staining | 15·80 | 2·64 |
| Normal | 10·75 | 1·32 |

abnormally staining cells. The autolytic tumour cells never reached the size of the abnormally staining cells. The difference in nuclear diameter between the largest autolytic cells and the latter cells is significant ($0{\cdot}001 > P$).

TABLE III.—*Percentage of Vitally Stained Cells and of Abnormally Staining Cells in Autolytic Tumour Related to Time*

| Time (hours) | Vitally stained cells (%) | Abnormally staining autolytic cells (%) |
|---|---|---|
| 0 | 0 | 0·0 |
| 3 | 0 | — |
| 4 | 3 | 20·0 |
| 48 | 92 | 49·2 |
| 72 | 100 | 56·6 |

*Vital staining*.—Table III shows that the number of stained cells increased with time and that the increase was not strictly parallel to the increase in the number of abnormally staining autolytic cells seen on the films.

### DISCUSSION

As Ehrlich's ascites carcinoma is a homograft one would expect to find evidence of injury to the cells following transplantation. Vital staining by Schrek's method (1936) is used extensively as evidence of cell death (Parker, 1961). However the assumption that all the cells taking up the stain are dead has been questioned (British Empire Cancer Campaign, Annual Report, 1951), and the conclusion reached was that while vital staining cannot be taken as certain evidence of cell death, all cells that do not take up the stain are viable.

With regard to Ehrlich's ascites carcinoma it has been shown that vital staining occurs just before the cell bursts and becomes pyknotic (King, Paulson, Puckett and Krebs, 1959). Cytological changes preceding vital staining were demonstrated in the autolytic tumour in the present work (Table III). These changes are thus an indication of cell injury but not of cell death.

The large cells found in the tumour in the experimental group are then probably, on the basis of their staining reaction, injured cells. Some of them, after prolonged exposure, will take up the vital stain. These injured cells were larger than the normal cells, and larger than the autolytic cells ever became (Table II). Increase in the size of tumour cells following injury is a common finding (Lumsden,

1931 ; Kidd, 1953 ; Klein and Forssberg, 1954 ; Flax, 1956 ; Green, Barrow and Goldberg, 1959) and it appears that the degree of swelling may vary with the different forms of injury (Koller and Casarini, 1952 ; Kalfayan and Kidd, 1953 ; Ahlström and Ising, 1955 ; King, Paulson, Puckett and Krebs, 1959).

Of the many cytological changes described following injury the changes in the large cells in the present experimental group were most like those described by Flax (1956) and Green, Barrow and Goldberg (1959) following treatment with a specific antitumour serum from a foreign host. Flax saw cytoplasmic changes and rupture of the cell membrane in air dried preparations, but he considered them to be artefacts. Green, Barrow and Goldberg describe similar cytoplasmic changes seen on phase contrast and electron microscopy. They did not see rupture of the cell membrane. In the present work the cytoplasmic changes were seen in wet as well as air dried preparations and the rupture of the cell membrane was seen to represent a stage in the disintegration of the cell.

The striking difference between the cells described by Flax and those reported here is that his cells formed ghost cells in the manner of the autolytic cells in this material. According to Green, Barrow and Goldberg more cytoplasmic swelling occurs in a low protein medium. As the protein content was probably much higher in Flax's experiment and in the autolytic tumour, in both of which a high proportion of the cells were degenerating, than in the tumours in this experimental group, the difference in medium could explain this discrepancy in the findings. Flax's findings also indicate that the formation of ghost cells is not related to the temperature of the medium.

The ghost cells in Flax's experiment were not preceded by pyknotic cells as was the case with the autolytic cells. It may be that in the autolytic cells degradation of the cytoplasmic and nuclear protein proceeded at the same rate while in his cells the cytoplasmic protein was denatured first and modified the disintegration of the nucleus. The relative lack of cytoplasmic protein in the large cells in the present experiment could account for the lack of modification in the nuclear degeneration.

Thus these large cells show changes characteristic of injury by specific antitumour antibody. The sequence of events suggests that the noxious agent acted on the surface exposed to the medium and points to the presence of a cytotoxic factor in the ascitic fluid ; this possibility is being investigated further.

The present work thus indicates that cellular injury does occur following the homotransplantation of Ehrlich's ascites carcinoma. It also shows that the number of cells injured varies from mouse to mouse (Fig. 1) as is to be expected as the mice used were heterozygous. The high positive correlation between the blood content of the tumour and the cellular damage supports the idea that they are both consequences of the same basic response, an immunity or homograft reaction.

### SUMMARY

The presence of injured cells in untreated Ehrlich ascites carcinoma is reported. The cytological changes were found to give a more delicate indication of cellular injury than vital staining. The morphological characteristics of the injured cells were similar to those described following treatment with specific antitumour serum, and indicate that the cells were damaged through a specific immune (homograft) reaction on the part of the host. This view is supported by the finding of a high positive correlation between the number of these cells present and the blood content of the tumour ascites, which is a measure of such a reaction (Hartveit, 1961*b*).

### REFERENCES

AHLSTRÖM, C. G. AND ISING, U. (1955)—*Acta path. microbiol. scand.*, **36**, 415.
BRITISH EMPIRE CANCER CAMPAIGN.—(1951) *Ann. Rep.*, **29**, 58.
FLAX, M. H.—(1956) *Cancer Res.*, **16**, 774.
GREEN, H., BARROW, P. AND GOLDBERG, B.—(1959) *J. exp. Med.*, **110**, 699.
HARTVEIT, F.—(1961*a*) *Brit. J. Cancer*, **15**, 336.—(1961*b*) *Ibid.*, **15**, 665.
KALFAYAN, B. AND KIDD, J. G.—(1953) *J. exp. Med.*, **97**, 145.
KIDD, J. G.—(1953) *Ibid.*, **98**, 565.
KING, D. W., PAULSON, S. R., PUCKETT, N. L. AND KREBS, A. T.—(1959) *Amer. J. Path.*, **35**, 1067.
KLEIN, G. AND FORSSBERG, A.—(1954) *Exp. Cell Res.*, **6**, 211.
KOLLER, P. C. AND CASARINI, A.—(1952) *Brit. J. Cancer*, **6**, 173.
LUMSDEN, T.—(1931) *J. Path. Bact.*, **34**, 349.
PARKER, R. C.—(1961) ' Methods of Tissue Culture '. New York (Paul B. Hoeber, Inc.), p. 282.
SCHREK, R.—(1936) *Amer. J. Cancer*, **28**, 389.

Since this paper went to press a further paper (Lindner, 1960) has come to the notice of the author. The morphological changes described in Ehrlich ascites carcinoma cells, following treatment with specific immune gamma globulin and supported by fluorescent antibody studies, appear identical to those described in untreated tumour in the present paper. This further supports the idea that these changes are the result of an immune reaction of the mice to the homografted tumour.

LINDNER, A.—(1960) *Amer. J. clin. Path.*, **34**, 426.